# Exogenous Application of dsRNA in Plant Protection: Efficiency, Safety Concerns and Risk Assessment

**DOI:** 10.3390/ijms25126530

**Published:** 2024-06-13

**Authors:** Mohammad Vatanparast, Lisa Merkel, Khalid Amari

**Affiliations:** Julius Kühn Institute (JKI), Federal Research Centre for Cultivated Plant, Institute for Biosafety in Plant Biotechnology, D-06484 Quedlinburg, Germany

**Keywords:** RNA interference, insect, pathogen, double-stranded RNA, formulation, risk assessment

## Abstract

The use of double-stranded RNA (dsRNA) for plant protection shows great potential as a sustainable alternative to traditional pesticides. This review summarizes the current state of knowledge on using exogenous dsRNA in plant protection and includes the latest findings on the safety and efficiency of this strategy. The review also emphasizes the need for a cautious and comprehensive approach, considering safety considerations such as off-target effects and formulation challenges. The regulatory landscape in different regions is also discussed, underscoring the need for specific guidelines tailored to dsRNA-based pesticides. The review provides a crucial resource for researchers, regulators, and industry stakeholders, promoting a balanced approach incorporating innovation with thorough safety assessments. The continuous dialog emphasized in this review is essential for shaping the future of dsRNA-based plant protection. As the field advances, collaboration among scientists, regulators, and industry partners will play a vital role in establishing guidelines and ensuring the responsible, effective, and sustainable use of dsRNA in agriculture.

## 1. Introduction

In agriculture, the constant struggle to develop resources to manage pests encompasses both insect pests and plant pathogens, which can also serve as carriers of severe diseases, has historically necessitated the extensive use of chemical pesticides for crop protection and disease management [1]. However, the widespread application of these chemicals has given rise to alarming rates of insecticide resistance, diminishing the efficacy of many common insecticides. Concurrently, public apprehensions concerning the environmental and health risks linked to these substances have intensified [2,3]. Consequently, within the frame of the Green Deal, the European Commission aims to adopt a proposal aiming at halving pesticide use by 2030. To address these challenges, there is an urgent call to explore safer and more precisely targeted alternatives for pest control.

**Figure 1:**
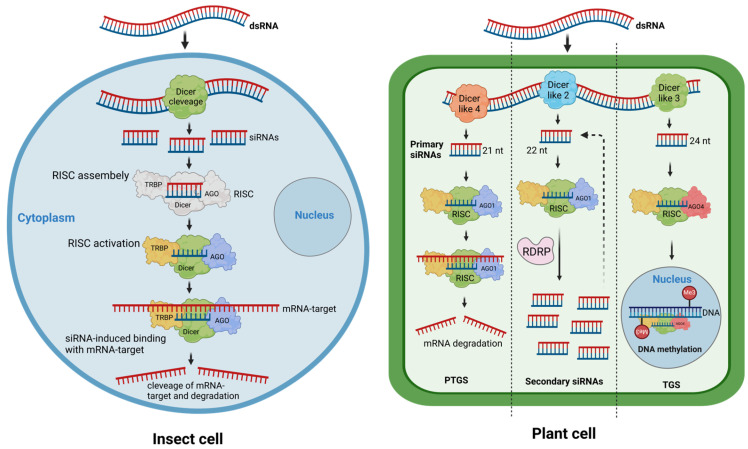
Schematic illustration of RNA interference pathway (exogenous) in insect (**left**) and plant cells (**right**). Double-stranded RNA molecules are processed by Dicer/Dicer-like proteins into siRNAs in both insect and plant cells. In insect cells, siRNAs bind to Argonaute (AGO) proteins to form RNA-Induced Silencing Complexes (RISCs), which then target complementary sequences on mRNA molecules, leading to post-transcriptional gene silencing (PTGS) through mRNA degradation. Meanwhile, in plant cells, distinct Dicer-like proteins (4, 2 and 3) generate siRNAs of varying lengths, which are loaded onto different AGO proteins (AGO1 and AGO4). These siRNAs play diverse roles, including cleavage of target transcripts, recruitment of RNA-dependent RNA polymerase (RDRP) for the generation of secondary siRNAs, or resulting in transcriptional gene silencing (TGS) in the nucleus and triggering de novo DNA methylation. Figure created with https://www.biorender.com/ on 28 March 2024.

Recent advancements in molecular biology and genetics have yielded innovative strategies for plant protection, among them the utilization of RNA interference (RNAi) technology. The development of RNAi was notably advanced in 1998, when a study showed that introducing dsRNA targeting a specific gene in the nematode *Caenorhabditis elegans* led to effective gene silencing [4]. Although similar RNAi effects had been observed earlier in plants, fungi, and nematodes [5], this research was crucial in establishing dsRNA as the mechanism behind the phenomenon. Further studies revealed that RNAi pathways are conserved across eukaryotic organisms and are integral to processes such as antiviral defense (via the small interfering RNA (siRNA) pathway), gene regulation (through the miRNA pathway), and genome protection against transposons (through the Piwi-interacting RNA pathway (piRNA)) [6]. Over the past 25 years, advancements in RNAi technology have driven progress in numerous biological disciplines, resulting in the development of RNAi-based treatments for human diseases and innovative approaches in agriculture for pest management and crop enhancement [7]. RNAi is an intrinsic mechanism where the ingestion or internalization of dsRNA, the key molecules of RNAi, triggers the precise regulation of gene expression by specifically targeting messenger RNAs (mRNAs) for degradation or translational inhibition. This process, commonly recognized as post-transcriptional gene silencing (PTGS), involves the transcription of mRNA from the DNA sequence of a gene. However, prior to the translation of this message into proteins by ribosomes, the mRNA undergoes obstruction or enzymatic degradation, a process guided by a specific non-coding siRNA (Figure 1), [8,9,10].

RNAi plays a pivotal role in gene regulation and defense against viral infections in plants [11,12]. While RNAi has been widely employed in animals, it has emerged as a promising tool for safeguarding plants against pests and pathogens, offering a sequence-specific method for suppressing the expression of particular genes. This approach can be finely tailored to the genetic diversity of each species, allowing for the precise targeting of genes crucial to the growth, development, or reproduction of pests, thus providing a selective approach to pest control that minimizes harm to non-target species [13]. RNAi not only safeguards crops but also holds promise for addressing broader agricultural challenges, including protecting crops against a wide array of pests and pathogens. Additionally, this technology offers several advantages over traditional pesticides, such as increased specificity, reduced environmental impact, and the ability to target genes that are traditionally challenging to address. In agriculture, where biotic stresses cause substantial losses on crop productivity, conventional breeding approaches face difficulties (e.g., long breeding programs and resistance breaking) in developing disease-resistant crops. As the world transitions towards sustainable crop production, RNAi emerges as an efficient and adaptable tool for combatting plant pests in a genetically specific manner. 

For employing dsRNA in pest control, there are two different delivery approaches. First, genetically modified (GM) RNAi crops have been engineered to stably express dsRNA, targeting the genes of specific pests. In this scenario, pests ingest the dsRNA while consuming the plant material [14,15,16]. In June 2017, US regulators granted approval for the first product of its kind in this category. This specific genetically engineered maize variety, known as MON87411, generates a dsRNA that targets the *Snf7* gene found in the western corn rootworm, *Diabrotica virgifera virgifera*, which belongs to the order Coleoptera of insects. SNF7 protein is a component of the ESCRT (Endosomal Sorting Complex Required for Transport)–III complex. This complex has been demonstrated to play a role in the sorting of transmembrane proteins during their transport along the endosomal-autophagic pathway, facilitating their eventual lysosomal degradation across various organisms [17,18,19,20,21]. Inhibiting the *Snf7* results in higher larval mortality and, as a result, diminished damage to the plant roots [22]. As a non-transgenic method, dsRNA molecules can be administered externally through a variety of techniques [23]. This includes their introduction into irrigation water or via trunk injections [24,25,26,27], as well as their incorporation into food baits. Innovative systems, such as the use of microorganisms, viruses, and nanocarriers [24,28,29,30,31] also facilitate their delivery. Second, dsRNA molecules can be applied topically in the form of spray-induced gene silencing (SIGS) [32]. The effectiveness of exogenously applied dsRNA-induced RNAi in plants may be influenced by various factors, including but not limited to the concentration and size of dsRNAs, application methods, delivery techniques, organ-specific activities within the plant or insect, and stability in adverse environmental conditions [33]. Ultimately, these factors play a crucial role in determining the rate of absorption and uptake of exogenous dsRNAs by targeting tissues, thereby initiating the RNAi response [34,35]. Moreover, there is well-documented evidence regarding the efficacy of dsRNA when externally administered to crops. The application methodology of these pesticides closely aligns with established practices in traditional pesticide application [32,36,37]. 

When it comes to using dsRNA for plant protection against insect pests, a set of challenges are present, particularly in the context of applying RNAi-based technology for pest control. In order to explore this topic, it is important to take into account two main factors. Firstly, the effectiveness of ingested dsRNA in inducing an RNAi response varies among different insect species [38]. Secondly, there is a growing concern that resistance may develop, not against specific dsRNA molecules but against elements of the dsRNA uptake and processing machinery [23]. A review discusses the physiological and adaptive factors that contribute to resistance to RNAi in insects, presenting a major challenge for the development of dsRNA-based pesticides [39]. Furthermore, it is important to consider a range of safety concerns. These include potential off-target effects, formulation challenges, potential impacts on epigenetic processes, and the potential for inducing the immune systems of non-target organisms (NTOs) [40]. As part of the regulatory process, dsRNA-based pesticide sprays must undergo environmental risk assessments (ERAs) before they are allowed for commercial release to mitigate environmental harm. These regulatory decisions are influenced by international discussions that aim to establish the necessary rules and data requirements, taking into account the unique mechanisms of dsRNA.

## 2. Exogenous dsRNA Application in Plant Protection

Exogenous application techniques include spraying, infiltration, injection, spreading, mechanical inoculation, and root/seed soaking. These methods have been used to deliver dsRNAs, siRNAs, and hairpins (hpRNAs) to plants to induce targeted gene silencing in plants, pathogens and pests [35] (Table 1). As it was mentioned in the previous section, several factors have the potential to impact the efficacy of RNA interference induced by exogenously applied dsRNA in plants. With current knowledge, the understanding of how these factors influence gene silencing through exogenous dsRNA remains limited [34,37]. The process for evaluating these RNA-based products closely resembles the assessment of conventional topical pesticides, where a solution containing siRNA or dsRNA is sprayed onto plant leaves or ingested by the target pests, and subsequent observations are made regarding its impact on insects [41].

Exogenous application of dsRNA through spray is effective for controlling pests and pathogens on different parts of plants, such as stems, foliage, and fruits. For instance, when the expression of two *ATPase* genes in *Diabrotica undecimpunctata* (Spotted cucumber beetle) and *Leptinotarsa decemlineata* (Colorado potato beetle, CPB) was suppressed, it led to a 40–50% reduction in insect survival [61]. One of the pioneering cases in the use of sprayable RNA molecules to combat insect pests involved the application of siRNA targeting the *acetylcholine esterase gene AchE2* in the diamondback moth (*Plutella xylostella*). The spraying of *Brassica* leaves with chemically synthesized siRNAs resulted in a high mortality rate for *P. xylostella* [62]. High-pressure spraying for siRNA effectively triggered local and systemic silencing of the green fluorescent protein (GFP) transgene in *Nicotiana benthamiana* plants [57].

Currently, various academic institutions and agribusiness giants, such as Syngenta, Bayer, Greenlight Bioscience, Corteva, BASF are actively working to advance the effective use of RNAi to protect crops from different insect species. Despite significant strides in comprehending the possibilities and constraints of this method, the availability of RNAi-related products in the market remains restricted. The most advanced approach for the application of insecticidal RNAi, both in terms of commercial deployment and regulatory evaluation, involves the *in planta* production of insect-specific dsRNA as a Plant-Incorporated Protectant (PIP). One of the first and most famous is maize which was produced by applying genetic modification (GM) to manage the western corn rootworm (WCR), *Diabrotica virgifera virgifera*. The registered GM maize product is identified as SmartStax Pro (MON87411) and is manufactured by Bayer.

Bayer’s “SmartStax Pro” maize is recognized as the pioneering RNAi-based pesticide product. It obtained regulatory approval from U.S. authorities in 2017 and, more recently, approved by the Chinese regulators in 2021. This maize variety combines the expression of the *Bacillus thuringiensis* (Bt) Cry3Bt1 toxin with glyphosate resistance and the presence of dsRNA that targets the *Snf7* gene of the WCR. RNA interference-mediated silencing of this gene, which plays a role in the transport of transmembrane proteins, results in the lethality of *D. v. virgifera*, ultimately reducing root damage [22]. The inclusion of the Bt-toxin enhances targeted pest control and resistance management [23]. This product is scheduled to be accessible to U.S. farmers in 2022 and to Canadian farmers from 2023 and now there are some online shops (for example: https://dynagroseed.com/agronomic-insights/bayers-smartstax-pro-now-in-dyna-gro-seed, accessed on 6 March 2024) where it can be bought. In Europe, “SmartStax Pro” maize has received authorization for all uses except cultivation [63]. Recently, a novel dsRNA insecticide named Calantha, designed by Greenlight Bioscience, with its active ingredient ledprona, a dsRNA targeting the *proteasome subunit β type 5* (*PSMB5*) gene of the Colorado potato beetle, has proven to be an effective tool for managing this pest through RNA interference [64]. In December 2023, the U.S. Environmental Protection Agency (EPA) registered Calantha for a three-year duration, consistent with the EPA’s usual approach for other novel biopesticide products. Earlier, in May 2023, the EPA approved an experimental use permit under the Federal Insecticide, Fungicide, and Rodenticide Act, allowing testing in 10 states. Subsequently, in September 2023, the EPA initiated a public comment period for the proposed registration [65]. 

The use of exogenous dsRNA was not limited to insect pests in plants but was also used for a wide range of plant pathogens. Several studies have reported successful applications of dsRNA in controlling plant diseases caused by viruses [60,66,67,68,69,70,71] and fungi [53,72,73]. Indeed, numerous studies have indicated that the activation of the RNA interference mechanism through the introduction of exogenous dsRNAs, siRNAs, or hpRNAs holds the promise of protecting plants against plant pathogens. The topical application of specific dsRNA to tomato plants significantly reduced the disease incidence caused by Tomato yellow leaf curl virus (TYLCV) [74]. Topical application of dsRNA targeting Papaya ringspot virus (PRSV) genes resulted in 100% resistance to PRSV-Tirupati and 94% to PRSV-Delhi in papaya, offering a non-transgenic control method for Papaya ringspot virus [69]. Another study demonstrated that the application of dsRNA molecules derived from Tobacco mosaic virus (TMV) p126 and coat protein genes effectively imparts resistance to TMV in tobacco plants [70].

RNAi-based control of fungal pathogens in plants was studied across a wide spectrum as well [75]. The foliar application of dsRNA targeting the *cytochrome P450* (*CYP3*) gene of *Fusarium graminearum* effectively inhibited fungal growth on directly sprayed leaves as well as on non-sprayed leaves of barley plants [53]. Spraying fruits, vegetables, and flowers with *Botrytis cinerea Dicer* or *Dicer-like1/2*-targeting dsRNAs or sRNAs leads to a significant reduction in gray mold disease [73]. The SIGS approach has shown remarkable success in countering a range of plant fungus pathogens, including, *B. cinerea* [56,73,76,77,78,79], *Sclerotinia sclerotiorum* [77], *F. asiaticum* [80], and more recently, *F. oxysporum* [54], *Phakopsora pachyrhizi* [55], *Aspergillus niger* and *Rhizoctonia solani* [78], and *Austropuccinia psidii* [81]. 

The findings from these research articles underscore the susceptibility of numerous pests, encompassing both insects and pathogens, to RNAi mechanisms, offering potential for the development of dsRNA-based biocontrol methods. Notably, a majority of the studies have focused on functional analysis or have been centered on crop plants. This highlights the significance of exogenous dsRNA application and the utility of SIGS as valuable tools. While SIGS has shown efficacy in laboratory settings, there remains a paucity of extensive, large-scale field trials. Additionally, critical aspects such as risk assessment and legislation governing SIGS use call for further improvement.

## 3. Risk Assessment and Regulatory Approaches

Double-stranded RNA molecules, essential to gene regulation and antiviral defense mechanisms, are prevalent in both plant and animal kingdoms. Consequently, they constitute natural components within the composition of various foods and feeds, boasting a lengthy history of human and vertebrate consumption with an established safety record [58]. The ubiquity of RNA in the environment and food sources has contributed to the robust physiological and biochemical barriers observed in mammals, further underscoring the negligible exposure to ingested dsRNAs. The gastrointestinal tract in humans and farm animals presents tough enzymatic degradation and cellular uptake barriers, thereby minimizing the potential impact of dsRNA ingestion [82,83]. A crucial factor to take into account is that dsRNA typically exhibits limited environmental persistence in soil, sediment, and water [84,85]. Notably, there is no scientific basis to suggest that the small RNAs present in foods, which originated from genetically modified plants expressing dsRNA, possess distinct properties or pose greater risks than their naturally abundant counterparts in conventional foods [63]. Similar to biopesticides, these factors might contribute to the reduced time and cost associated with gathering data for registering a dsRNA-based pesticide compared to a conventional pesticide, with a timeframe of 4 years as opposed to 12 years and costs amounting to USD 3–7 million in contrast to USD 280 million [86]. The available studies strongly suggest that systemic absorption of intact dsRNA from RNAi-based pesticides in humans and vertebrates is highly improbable, a notion supported by the extensive history of safe dsRNA consumption in natural foods [12,84,85]. The presence of biological barriers in mammals, combined with swift metabolisms and clearance mechanisms, further minimizes potential risks [12,87]. Although regulatory procedures for dsRNA-based agricultural products lack standardization, the current framework designed for small-molecule agrochemicals is deemed appropriate, reflecting the evolving regulatory landscape [87]. 

**Figure 2:**
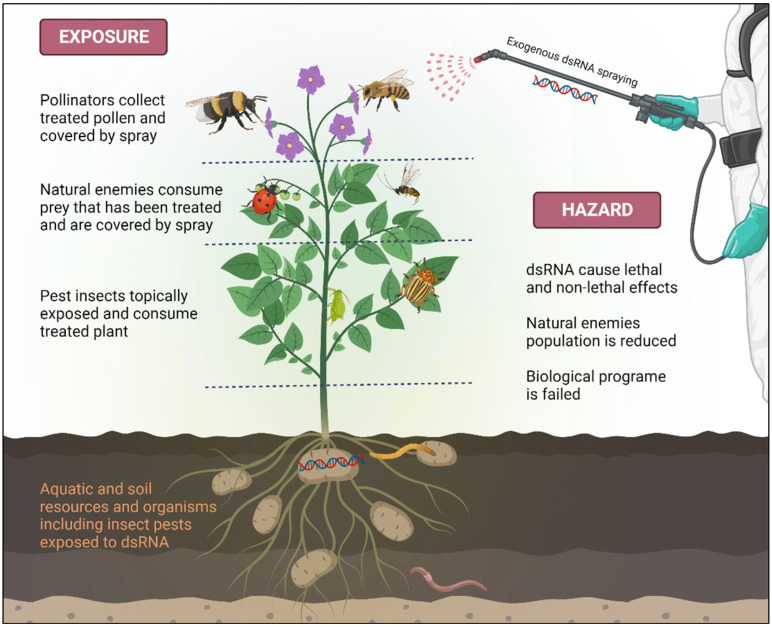
Exposure pathways of target and non-target organisms to applied dsRNA, including direct contact during feeding or grooming and topical application. This introduces plausible routes of exposure. Natural enemies may also encounter dsRNA while feeding on pests, potentially affecting biological pest control. Figure created with https://www.biorender.com/ on 23 November 2023.

When applying RNAi-based products in the field against insect pests, it is essential to consider that the impact of dsRNA on invertebrates differs from its effects on vertebrates. Both the target pest and non-target organisms, along with the surrounding environment, may be exposed to dsRNA (Figure 2). This exposure can occur through direct contact during feeding on treated plants, absorption or grooming after topical application, or contact with dsRNA present in the environment, such as soil or water. Additionally, natural enemies may be exposed to dsRNA by consuming pests that have been previously exposed to dsRNA [88] (Figure 2). The extent of exposure for NTOs is influenced by various factors, including application rate, timing, method, frequency, off-site movement of dsRNA, as well as the stability and persistence of the dsRNA [23]. Despite the inherent low hazard of active dsRNA molecules, recognizing potential risks in all technologies emphasizes the necessity for a comprehensive risk analysis. This analysis guides the development of robust, science-based safety practices designed to effectively minimize or mitigate any conceivable adverse effects [89]. The environmental stability of the active dsRNA component is notably low, with microbial nucleases, UV radiation, and runoff from dew and rain significantly restricting its availability to pests [84,85]. Hence, the successful implementation of dsRNA in an RNAi-based approach demands the development of stabilizing formulations. These formulations play a dual role by not only enhancing cellular internalization of dsRNA but also protecting it from nucleolytic degradation, thereby optimizing its overall delivery to the target pest [49,63,90]. Considering the potential influence of formulations on the environmental persistence of dsRNA and human exposure pathways, it becomes imperative to incorporate formulations into the risk assessment of exposure, necessitating a case-specific evaluation [85]. Moreover, it is crucial to recognize that formulations themselves may pose environmental and non-target organism risks, justifying a comprehensive assessment of their impact in addition to dsRNA assessment [63]. 

As SIGS-based products entering the market, it is imperative to establish a comprehensive regulatory framework and explicit guidelines for the risk assessment and registration of these innovative plant protection products. Various considerations must be taken into account concerning the data required to substantiate pesticides or plant protection products (PPPs) based on dsRNA, given their specific mode of action. In this section, we draw attention to certain factors that could influence data requirements for risk assessment and decision-making. 

The regulatory landscape for dsRNA-based pesticides varies across regions, with notable distinctions in the USA, Australia, and Europe. In the USA, these products are considered biochemical pesticides, necessitating Environmental Protection Agency (EPA) approval under the Federal Insecticide, Fungicide, and Rodenticide Act (FIFRA) and the Federal Food, Drug, and Cosmetic Act (FFDCA) [91,92]. Australia classifies them as agricultural chemical products, regulated by the Australian Pesticides and Veterinary Medicines Authority (APVMA), with the Office of the Gene Technology Regulator (OGTR) considering SIGS applications non-GMO under specific conditions [63].

The regulatory framework for RNAi in plant production in the European Union (EU) is primarily outlined in Directive 2001/18/EC (EU, 2001) and Regulation (EC) 1829/2003 [93]. When HIGS-based products are intended for food and feed, they fall under Regulation (EC) 1829/2003 [93]. The classification as genetically modified organisms (GMOs) is determined by whether the products contain living organisms or only purified molecules. If they contain viable GMOs, they must be authorized according to EC Directive 2001/18 [93]. If no GMOs are used or it is ensured that they have all been inactivated, the SIGS-based products should not be considered GMOs, and their registration might follow the same regulatory framework as classical synthetic pesticides under Regulation (EC) 1107/2009 [94]. In this case, the approval procedure for dsRNA as a pesticide in Europe follows a two-step process outlined in Regulation (EC) No. 1107/2009. Initially, the active substance undergoes evaluation in a comprehensive EU-wide process led by the European Food Safety Authority (EFSA) and endorsed by the EU Commission. The second step involves the assessment of the pesticide product by individual Member States (MS). A zonal approach, introduced to streamline the authorization process, divides the EU into three zones: northern, central, and southern. In each zone, a designated Member State (zonal rapporteur Member State) (zRMS) evaluates the pesticide risk for the entire zone, with MS obliged to adhere to the zRMS’s conclusions. However, MS retains the authority to identify national specificities and determine risk management options [95].

Double-stranded RNA is considered a new class of active substances, and its assessment might follow the data requirements for active substances described in Regulation (EC) 283/2013 [96]. However, specific data requirements for SIGS-based PPPs have not been explicitly specified. The assessment is conducted using the same requirements as for chemical PPPs outlined in Regulation (EC) 284/2013 [97] and Regulation (EU) 546/2011 [98]. Adaptations of data requirements may be considered on a case-by-case basis, and guidance documents from the Organization for Economic Co-operation and Development (OECD), Plant Protection Organization (EPPO), and EFSA offer support for the methodological requirements for risk assessment [95]. In the EU, the regulatory focus was initially on chemicals as active substances, but Article 77 of Regulation (EC) No 1107/2009 [94] allows for the adoption or amendment of guidance documents for other product classes, like dsRNA. The EU has already implemented specific adaptations for microorganisms, pheromones, and botanicals, but as of now, there are no specific guidance documents defining the data requirements for the authorization of dsRNA-based PPPs. A comprehensive examination of the current status of these considerations is provided in the OECD Working Paper titled “Considerations for the Environmental Risk Assessment of the Application of Sprayed or Externally Applied ds-RNA-Based Pesticides” [40], which provides insights into environmental risk assessments for dsRNA-based pesticides. The document recommends considerations for assessing off-target effects. However, specific guidance documents defining data requirements for the authorization of dsRNA-based PPPs are not yet in place, and the implementation of such rules may take some time. 

In summary, PPPs based on dsRNA are currently categorized as chemical PPPs in the EU regulatory framework. Specific guidance documents for dsRNA-based PPPs are under consideration, and adaptations to data requirements may be introduced in the future based on the evolving understanding of this new class of active substances.

## 4. Safety Concerns of dsRNA Application

The application of double-stranded RNA in pest management, particularly through RNA interference, introduces potential safety concerns that must be thoroughly examined. One of the primary challenges associated with dsRNA lies in its off-target effects, wherein unintended gene silencing of non-target genes may occur. This can lead to undesirable outcomes, such as developmental abnormalities or diminished growth in non-target organisms. Research in mammalian cells has unveiled that siRNA/dsRNA not only induces non-specific off-target effects, such as an immune response [99], competition between siRNA and miRNA [100], and downstream effects like alterations in the expression of non-target genes [101], but also specific off-target effects. These specific effects arise due to siRNA hybridizing with unintended mRNA, leading to the degradation of unrelated transcripts [102,103,104]. 

To address this issue, several strategies have been developed, such as the use of dsRNA with high specificity, the selection of target genes with minimal homology to other NTOs genes, and the use of delivery methods that minimize the spread of dsRNA to non-target tissues. These concerns can be broadly categorized into sequence-specific and sequence-unspecific effects [19]. Despite the inherent specificity of RNAi for target organisms, the existence of partial homology in dsRNA sequences raises the risk of off-target effects [58,101]. Bioinformatics tools assist in identifying potential off-target sequences, but knowledge gaps hinder accurate predictions. siRNA variability, differences in mismatch tolerance, and the impact of mismatch location and type add complexity to the sequence-specific effects. While bioinformatics aids in selecting non-target species, it cannot replace empirical approaches, emphasizing the need for a comprehensive risk assessment involving diverse organisms [63,105]. 

The theoretical risk of saturating the RNAi pathway at high dsRNA concentrations is a concern, though evidence in arthropods is currently lacking [106]. Additionally, dsRNA exposure may stimulate an immune response independent of sequence specificity. Recognition of dsRNA by innate immune system receptors can trigger immune responses, influencing insect performance [89]. The activation of the innate immune system has been investigated through in vitro methodologies employing transfection reagents and elevated RNA concentrations. Additionally, animal models have been utilized in certain instances. These immune responses are facilitated by receptors that engage with double-stranded RNA, notably Toll-like receptors (TLR3, TLR7, TLR8), and enzymes such as the double-stranded RNA-binding protein kinase PKR, as well as the RNA helicases RIG-I and MDA-5 [87,107]. It has been demonstrated that the application of double-stranded RNA (dsRNA) in plants can stimulate an immune response through pattern-triggered immunity (PTI), which operates independently of RNAi. These findings should be taken into account when conducting risk assessments [108].

Inside plant cells, there is evidence suggesting that dsRNAs undergo multiple processing steps. These steps not only activate the RNA interference machinery but may also include transitive and systemic silencing, along with potential involvement in epigenetic modifications [35,109]. Research on RNA interference has established a connection between RNAi and RNA-directed DNA methylation (RdDM), suggesting that siRNAs play a role in initiating and directing cytosine methylation [110]. It was observed that the application of exogenous dsRNAs targeting enhanced green fluorescent protein (EGFP) and neomycin phosphotransferase–II (NPTII) in transgenic Arabidopsis led to a significant enhancement in cytosine DNA methylation, a universally recognized epigenetic mechanism [111]. 

The extent and likelihood of these sequence-unspecific effects require further exploration. Unlike conventional pesticides, RNAi-based products may exhibit a delayed efficacy, requiring an extended observation period [23,92]. Evaluation should encompass non-lethal phenotypes and life cycle analyses, going beyond mere mortality rates.

The successful implementation of dsRNA in a SIGS approach relies on the use of stabilizing formulations, such as nanoparticles, liposomes, and chitosan [112]. These formulations not only enhance cellular internalization and protect dsRNA from nucleolytic degradation but also influence its stability, bioavailability, and toxicity [49,63,90]. The choice of formulations can affect the persistence of dsRNA in the environment and human exposure pathways, necessitating a case-by-case assessment to evaluate their influence on exposure risks. Therefore, a comprehensive understanding of the environmental and human implications of dsRNA formulations is crucial, considering both their efficacy in pest management and potential consequences for safety. 

Despite the emergence of RNAi-based products, a consensus on data requirements for risk assessment is yet to be established. The selection of appropriate non-target species should be based on sensitivity, relevance, and the availability of reliable test protocols [23]. This approach ensures a tailored risk assessment that considers the unique characteristics of dsRNA-based products.

In conclusion, while the potential benefits of dsRNA application in pest management are significant, an in-depth understanding of safety considerations is imperative. Addressing both sequence-specific and sequence-unspecific concerns through a combination of bioinformatics tools, empirical studies, and continuous input from fundamental research ensures a comprehensive evaluation of the safety profile of dsRNA-based products in diverse ecological contexts.

## 5. Efficiency and Perspectives of RNAi in Crop Protection

RNA interference technology has emerged as a powerful tool in functional genomics studies over the past two decades. Recent advancements have positioned RNAi as a potential solution to global agricultural challenges posed by insects and pathogens in a sustainable manner [113]. The application of RNAi, particularly double-stranded RNA, has received significant attention and funding support. While transgenic crops expressing dsRNA offer improved effects, they face regulatory hurdles as genetically modified products. The commercialization of SmartStax Pro maize represents a positive development, yet challenges persist in extending transgenic technology [114]. Calantha, a newly formulated sprayable biopesticide containing dsRNA (active ingredient ledprona), is currently registered in the United States, reflecting the continuous progress in the creation of advanced dsRNA-based pesticides [42,64]. To broaden the scope, there is a need for the development of new chloroplast transformation protocols for major crops. The utilization of chloroplast transformation presents a promising avenue for enhancing the efficacy of RNAi as a pest control strategy in major crop plants. Unlike plant cell nuclei, chloroplasts lack RNAi machinery, thus providing a unique environment for the expression of dsRNA without triggering off-target effects on the host plant [115]. Recent studies, such as the work by Zhang et al. [116] in potato, have demonstrated the feasibility of this approach. By expressing dsRNA targeting specific insect genes in chloroplasts, transplastomic plants accumulated high levels of dsRNA in leaves, resulting in significantly enhanced RNAi efficacy against the target pest, *Leptinotarsa decemlineata*. Additionally, the application of chloroplast-mediated RNAi demonstrated notable impacts on *Helicoverpa armigera* [115] and *Manduca sexta* [117]. Importantly, chloroplast-mediated RNAi capitalizes on the feeding behavior of these pests, which involves the ingestion of solid plant tissues, thereby facilitating the release and uptake of dsRNA. This strategy has the potential to expand RNAi-based pest management to diverse crops, promoting sustainable agriculture.

Additionally, RNAi products, delivered through various methods such as foliar spray, irrigation, and trunk injection, hold promise for enhancing insecticidal activity.

Bacteria-based expression of dsRNA is considered a cost-effective method, with biotech companies investing in this approach to produce affordable dsRNA for both small and large farms. Public concerns about dsRNA specificity, the fate of nanoparticle/dsRNA formulations in the environment, and the impact on non-target organisms underscore the importance of addressing ethical and ecological considerations. Looking ahead, RNAi holds great promise in pest control due to its species-specific action and natural degradation in the environment. While transgenic GM-maize has gained approval, non-transgenic sprayable RNAi offers an alternative, particularly in regions with stringent regulations on GM crops. The rapid degradation of dsRNA may be less problematic in greenhouse settings, making sprayable RNAi products competitive in high-value crop applications. Despite the potential, RNAi faces challenges. Its comparably slow action poses limitations in situations where swift insect control is essential. Cost remains a factor, although production advancements have lowered costs significantly. The balance for the future of sprayable RNAi in agriculture is uncertain, and it may find niche applications or serve as a resistance-management tool in specific settings.

In summary, RNAi technology represents a versatile option for pest and disease control in crops. Ongoing developments in production cost reduction and stability enhancement, particularly through encapsulation strategies, are paving the way for broader adoption. The regulatory landscape appears optimistic, considering the economic and environmental advantages coupled with low risks to human health. Interdisciplinary approaches, such as combining RNAi with elicitation and metabolic control methods, showcase the potential for future innovations in crop protection. The continued reduction in production costs through biotechnological advancements is crucial for the widespread and profitable implementation of RNAi technology on a large scale.

## Figures and Tables

**Table 1:** Examples of applications of exogenous dsRNAs in different targets.

Group	Target Organism	Gene	Plant	Application Method	Formulation	dsRNA Amount	Efficacies of Formulation	Durability	Reference
Insects	*Leptinotarsa decemlineata*	Proteasome subunit beta 5	*Solanum tuberosum*	Sprayable dsRNA-biopesticide	Unknown	25 × 10^−6^ g/L	90% mortality after 6 days of exposure	Increased mortality up to 9 d. after treatment	[42]
	*Acyrthosiphon* *pisum*	vATPase	*Vicia faba*	Feeding dsRNA via artificial diet	Naked dsRNA	1000 ng/μL	High levels of mortality	6–7 days	[13]
	*Henosepilachana vigintioctopunctata*	Ecdysone receptor	*Solanum tuberosum*	dsEcR-*E. coli* directly sprayed to the foliage	*Escherichia coli* HT115	0.5 μg/mL	40% reduced larvae pupation, 60% showed defective phenotype	Up to 10 days	[43]
	*Sitobian avenae*	Salivary sheath protein	*Hordeum vulgare*	Foliar spray on leaves	Naked dsRNA	20 ng/μL	60% reduction in transcript levels of target gene and disease resistance	Effect: 5 days; dsRNA detectable: 3–9 days	[44]
	*Helioverpa armigera*	Juvenile hormone methyltransferase, Acetylcholine esterase	*Cicer arietinum*	Hand-held mist sprayer	Chitosan nanoparticles	200 μg	100% insect mortality	Up to 5 days	[45]
	*Myzus persicae*	MpC002, Rack1	*Nicotiana benthamiana* and *Arabidopsis thaliana*	Host induced gene silencing via transgenic plants)	Naked dsRNA produced by transgenic plants	15–20 ng	Target gene expression knockdown by 60%, reduced aphid fecundity	17 days after feeding	[46]
	*Aphis glycines*	TREH, ATPD, ATPE, and CHS1	*Glycine max*	Transdermal dsRNA delivery system	Star polymer (SPc) nanoparticles	500 ng/μL SPc and dsRNA	Significant gene silencing 48 h after treatment.	Significant mortality increase 4 day after treatment	[47]
	*Blattella germanica*	α-Tubulin	-	Oral delivery	Liposomes	0.25 μg/μL of dsRNA lipoplexes	24–60% increased mortality	Increase of mortality for 16 days	[48]
	*Spodoptera exigua*	Chitin synthase B	-	In vivo feeding bioassays	Guanidine-containing polymers	500 ng/μL	Mortality with formulation: 53%; without formulation: 16%	Prevention of larvae pupation up to 15 days	[49]
	*Sogatella furcifera*	Vacuolar-type (H+)-ATPase	Rice	Spraying and injection	SPc	1000 ng/μL	More than 97%	7 days post treatment	[50]
	*Bemisia tabaci*	Toll-like receptor 7	*Hibiscus rosasinensis*	Recombinant *Isaria fumosorosea*, leaf immersion method	Fungi conidia	2 × 10^7^ spore/mL	90.33%	8 days after treatment	[51]
	*Tribolium castaneum*	BiP, Armet	Insect rearing on wheat flour	Diet containing dsRNA-BAPCs nanoparticles	Branched amphiphilic peptide capsules (BAPCs)	500 ng	Ingestion of dsRNA/ BAPC complexes increased mortality from 40% to 75%	Up to 40 days	[52]
Fungi	*Fusarium garminearum*	Cytochrome P450 lanosterol C-14α-demethylases	*Hordeum vulgare*	Foliar spray on leaves	Naked dsRNA	20 ng/μL	Reduced transcript levels of target gene of 75%	Effect detectable 6–8 post spraying; unprocessed ds/siRNA accumulation detectable 24 h post spraying	[53]
	*Fusarium oxysporum*	CYP51, chitin synthase 1, Elongation factor 2	*Solanum lycopersicum*	Topical delivery	Layered double hydroxide clay nanosheets	100 μg/mL	Reduced fungal growth; 93% reduction in transcript abundance	Up to 8 days	[54]
	*Phakopsora pachyrhizi*	Acetyl-CoA acyltransferase 40S ribisomal protein S16, Glycine cleavage system H-protein	*Glycine max*	Spraying	Diethylpyrocarbonate	20 μg	<73% reduction in fungal infection-symptoms, 75% reduction in biomass accumulation	2 weeks	[55]
	*Botryotiania fuckeliana*	Chitin synthase class III, DCL 1, DCL2	*Fragaria ananassa*	Topical spray application	*Escherichia coli*-derivedanucleated minicells	125–1000 ng/mL	Knockdown of target genes, leading to significant reduction in fungal growth	Up to 12 days	[56]
Plants	*Nicotiana benthamiana*	Green flourecent protein (*GFP*), 21–24 nt	*Nicotiana benthamiana*	High-pressure spraying method	Naked dsRNA	10 µM	Local and systemic silencing	20 dpa	[57]
	*Nicotiana benthamiana*	GFP, siRNA loading	*Nicotiana benthamiana*	1-mL needleless syringe	DNA nanostructures	100 nM	40–59% reduction in mRNA- and protein-level	Up to 7 days	[58]
Viruses	Bean common mosaic virus	Nuclear inclusion b-protein	*Nicotiana benthamiana*	Manual inoculation via coborundum	Layered double hydroxide clay nanosheets	100 μg	Reduction of infection up to 45%	Up to 10 days	[59]
	Sugarcane Mosaic virus	Coat protein	*Zea mays*	Spraying bacteria-produced dsRNA	*Escherichia coli* HT115	3 μg/L	Total inhibition of virus infection	30 dpi	[60]
	Pepper mild mottle virus (PMMoV) and cucumber mosaic virus (CMV)	replicase gene of PMMoV14; 2b suppressor of plant antiviral RNA silencing defense encoded by CMV25	*Arabidopsis thaliana*, *Vigna unguiculata*, *Nicotiana tabacum*	Topical application as spray	Layered double hydroxide (LDH) clay nanosheets (BioClay)	1.25 μg	spray extended virus protection from 5–7 days to <20 days	Significantly increased resistance to virus infection: up to 20 days post spraying	[37]
